# Use of single-chamber atrial pacemakers post-DANPACE: an analysis using national data

**DOI:** 10.1093/europace/euae290

**Published:** 2025-01-28

**Authors:** Paul A Scott, Ian J Wright, Daniel I Bromage, Chris Plummer, Mark de Belder, Mark Dayer, Francis D Murgatroyd

**Affiliations:** Department of Cardiology, King’s College Hospital NHS Foundation Trust, Denmark Hill, London SE5 9RS, UK; King’s British Heart Foundation Centre of Research Excellence, King’s College London, 125 Coldharbour Lane, London SE5 9NH, UK; NHS Arden and GEM Commissioning Support Unit, National Institute for Cardiovascular Outcomes Research (NICOR), Westgate House, Market Street, Warwick CV34 4DE, UK; Department of Cardiology, Hammersmith Hospital, Imperial College Healthcare NHS Trust, London, UK; King’s British Heart Foundation Centre of Research Excellence, King’s College London, 125 Coldharbour Lane, London SE5 9NH, UK; Department of Cardiology, Freeman Hospital, The Newcastle Upon Tyne Hospitals NHS Foundation Trust, Newcastle upon Tyne, UK; NHS Arden and GEM Commissioning Support Unit, National Institute for Cardiovascular Outcomes Research (NICOR), Westgate House, Market Street, Warwick CV34 4DE, UK; NHS Arden and GEM Commissioning Support Unit, National Institute for Cardiovascular Outcomes Research (NICOR), Westgate House, Market Street, Warwick CV34 4DE, UK; Cardiovascular Research Institute, Mater Private Network, Dublin, Ireland; Faculty of Health, University of Plymouth, Plymouth, UK; Department of Cardiology, King’s College Hospital NHS Foundation Trust, Denmark Hill, London SE5 9RS, UK; King’s British Heart Foundation Centre of Research Excellence, King’s College London, 125 Coldharbour Lane, London SE5 9NH, UK; NHS Arden and GEM Commissioning Support Unit, National Institute for Cardiovascular Outcomes Research (NICOR), Westgate House, Market Street, Warwick CV34 4DE, UK

**Keywords:** Complication, Pacemaker, Reoperation, Risk

In patients with sinus node disease (SND) and intact atrio-ventricular conduction, normal physiologic cardiac activation may be achieved by single-chamber atrial or dual-chamber pacing. Although early studies suggested a benefit of single-chamber atrial over dual-chamber pacing in patients with SND, the Danish Multicentre Randomized Trial on Single Lead Atrial vs. Dual Chamber Pacing in Sick Sinus Syndrome (DANPACE), demonstrated no mortality benefit of single-chamber atrial pacing but a two-fold increase in risk of reintervention compared to dual-chamber devices.^1–3^ As a result of these data guidelines recommend single-chamber atrial PPMs as a second line option in SND.^3^

It is unknown how often and in which patients single-chamber atrial PPMs are now used. Furthermore, it is unclear if the high risk of reintervention found in DANPACE is mitigated by their judicious use in selected patients outside of clinical trials. This study aimed to explore temporal trends in the use of single-chamber atrial PPMs and evaluate the association between pacemaker type and reintervention risk in routine clinical practice.

NHS hospitals in England are contractually required to submit details of device procedures to the National Institute for Cardiovascular Outcomes Research (NICOR). We used the NICOR database to identify patients receiving their first transvenous single- or dual-chamber PPM implant in England over 5 years (April 2014—March 2019).

We evaluated temporal changes in device use and the association between device type and reintervention risk. Our primary end-point was device reintervention at 1-year. We also evaluated any reintervention during the study period. Reinterventions comprised: lead revision (with or without a new generator), generator change alone, upgrade, downgrade, explant, pocket revision or haematoma evacuation.

To enable at least 12 months follow-up for all implants, the database was searched from April 2014 to March 2020. The COVID-19 pandemic did not impact cardiac procedures in the UK until after the study period.

Categorical variables were compared using the χ^2^ test and trends across categories using the Mantel–Haenszel test for trend. We performed survival analysis for any device reintervention. Patients were grouped by device type and compared using the log-rank test.

We used multilevel logistic regression to evaluate associations between device type and reintervention. We first explored the relationship in an unadjusted model. We then generated a multivariable model adjusted for implant year, patient age and gender, and operator/hospital characteristics (including operator/hospital volume, operator seniority and hospital type).

During the study, 149 631 first transvenous PPM implants were reported by NHS hospitals in England. We excluded 25 313 cases due to missing data, leaving 124 318 PPM implants for analysis: 218 single-chamber atrial, 32 403 single-chamber ventricular and 91 697 dual-chamber PPM. The median patient age was 79.9 years and the majority were male (60.4%). Of the patients where the indication for a device was clear (*N* = 114 974), most were implanted for atrioventricular block ± SND (74.7%), with a minority implanted for SND alone (25.3%).

A small number of single-chamber atrial pacemakers were implanted each year, though as a proportion of annual PPM implants this number gradually decreased during the study period (0.3% of implants in 2014/15 to 0.1% in 2018/19; *P* < 0.001 for trend).

Proportionally, the use of single-chamber atrial devices was highest in younger patients and decreased with advancing implant age (3.3% < 30 years vs. 0.1% > 70 years; *P* < 0.001 for trend) (*Figure 1*). Compared to other device types, single-chamber atrial devices were more likely to be implanted in women (*P* < 0.001).

**Figure 1:**
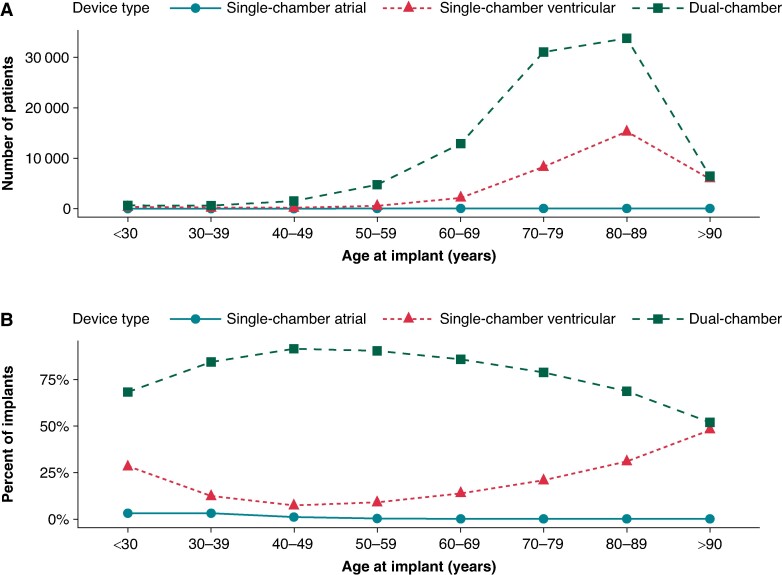
Choice of PPM type and patient age for first PPM implants (2015–2019) (*N* = 124 318). (*A*) Number of implants for each device type, with patients grouped by age at implant. (*B*) Proportion of implants for each device type, with patients grouped by age at implant.

The 1-year reintervention rate was higher for single-chamber atrial devices (10.6%) than dual-chamber devices (4.3%) and single-chamber ventricular PPMs (3.7%) (*P* < 0.001). The higher reintervention rate in single-chamber atrial devices was driven by both higher rates of lead revision (5.5 vs. 3.1%, *P* = 0.04) and device upgrade (2.8 vs. 0.5%, *P* = 0.001), compared to other device types.

These findings persisted when follow-up was extended to the duration of the study period (mean 3.2 ± 1.5 years): reintervention was higher for single-chamber atrial devices (16.1%) than dual-chamber devices (6.2%) and single-chamber ventricular PPMs (5.2%) (*P* < 0.001). Furthermore, when we included only patients implanted for SND (*n* = 29 180) the results were similar for both 1-year and the longer follow-up period.

Using survival analysis yielded similar results. For the total cohort (*N* = 124 318) the reintervention rates at 1-, 3- and 5-years were for 10.6, 13.6 and 19.5% for single-chamber atrial PPM, 4.3, 6.0 and 7.4% for dual-chamber PPM and 3.7, 5.0 and 6.0% for single-chamber ventricular PPM (*P* < 0.001). When only patients implanted for SND (n = 29 180) were included the rates were 11.1, 14.2 and 20.4% for single-chamber atrial PPM, 4.1, 5.2 and 6.2% for dual-chamber PPM and 4.8, 5.5 and 6.2% for single-chamber ventricular PPM (*P* < 0.001).

In an unadjusted model including all cases (*N* = 124 318), single-chamber atrial PPMs were associated with a higher risk of 1-year reintervention than dual-chamber PPMs (odds ratio [OR] 2.58; 95% confidence intervals [CI] 1.66–4.00; *P* < 0.001) (*Table 1*). There were similar findings in a model adjusted for basic patient demographics and operators/institution volume/characteristics. Furthermore, when only patients implanted for SND alone were included (*N* = 29 180), there were similar findings (*Table 1*).

**Table 1:** Association between device type and 1-year reintervention following first PPM implant

	1-year reintervention	
	OR	*P* value
All cases		
Unadjusted (*N* = 124 318)		
Dual-chamber	1 [Reference]	–
Single-chamber ventricular	0.84 (0.79–0.90)	<0.001
Single-chamber atrial	2.58 (1.66–4.00)	<0.001
Adjusted^a^ (*N* = 123 974^b^)		
Dual-chamber	1 [Reference]	–
Single-chamber ventricular	0.96 (0.89–1.03)	0.22
Single-chamber atrial	2.07 (1.31–3.25)	0.002
Only SND		
Unadjusted (*N* = 29 180)		
Dual-chamber	1 [Reference]	–
Single-chamber ventricular	1.15 (0.94–1.41)	0.16
Single-chamber atrial	2.85 (1.82–4.46)	<0.001
Adjusted^a^ (*N* = 29 124^c^)		
Dual-chamber	1 [Reference]	–
Single-chamber ventricular	1.36 (1.10–1.67)	0.004
Single-chamber atrial	2.33 (1.45–3.74)	<0.001

OR, odds ratio. SND, sinus node disease

^a^Adjusted for implant year, patient age (categorical), patient sex, hospital annualized implant volume and characteristics (whether implanting hospital performed on-site cardiothoracic surgery and whether the hospital implants complex devices), supervising consultant annualized implant volume and seniority, and supervising consultant characteristics (practicing cardiac electrophysiologist, complex device implanter, initial country of medical qualification).

^b^Missing data in 344 patients.

^c^Missing data in 56 patients.

These findings demonstrate that although there has been a significant reduction in the use of single-chamber atrial PPM, they are still implanted. Furthermore, even when used sparingly in selected patients, the need for reintervention is higher than dual-chamber devices. These findings are consistent with DANPACE and support the guideline position concerning the use of single-chamber atrial devices.^1,3^

We found single-chamber atrial devices were more likely to be implanted in younger patients and women. The use in younger patients, presumably to avoid lead-related complications, is understandable and consistent with the guidelines.^3^ The reasons for their greater use in female patients is unclear, though include the challenges of obtaining venous access in smaller patients, who are more likely to be female.

Our study has important limitations that need to be considered when interpreting our results and may lead to uncertainty concerning its generalizability. First, data are not available concerning confounding factors including patient comorbidity, frailty, and potential problems with venous access. Second, although the study was large, the number of single-chamber atrial devices was low and consequently likely a highly selected group of patients.

## Data Availability

The data underlying this article cannot be shared publicly. The data are from NHS patients, collected by NICOR and controlled by NHS England. As a consequence, there are strict rules, administered by NHS England, governing use of the data and it is not possible to share it with third parties.
